# Circularly polarised phosphorescent photoluminescence and electroluminescence of iridium complexes

**DOI:** 10.1038/srep14912

**Published:** 2015-10-08

**Authors:** Tian-Yi Li, Yi-Ming Jing, Xuan Liu, Yue Zhao, Lin Shi, Zhiyong Tang, You-Xuan Zheng, Jing-Lin Zuo

**Affiliations:** 1 State Key Laboratory of Coordination Chemistry, Collaborative Innovation Center of Advanced Microstructures, School of Chemistry and Chemical Engineering, Nanjing University, Nanjing 210093, P. R. China; 2 National Center for Nanoscience and Technology, 11 Beiyitiao, Zhongguancun, Beijing, 100190, P. R. China

## Abstract

Nearly all the neutral iridium complexes widely used as dopants in PhOLEDs are racemic mixtures; however, this study observed that these complexes can be separated into stable optically active Λ and ∆ isomers and that their chirality is an intrinsic property. The circularly polarised phosphorescent photoluminescence (CPPPL) signals of Λ/Δ isomers are perfect mirror images with opposite polarisation and equal intensity exhibiting a “handedness” for the polarisation. For the first time, we applied the Λ/Δ iridium isomers as emitters in OLEDs, and the circularly polarised phosphorescent electroluminescence (CPPEL) spectra reveal completely positive or negative broad peaks consistent with the CPPPL spectra. The results demonstrate that the Λ/Δ isomers have potential application for 3D OLEDs because they can exhibit high efficiency and luminance, and 3D display technology based on circularly polarised light is the most comfortable for the eyes.

Circularly polarised light (CPL) has potential application in various fields, and control of the polarisation of light is essential for optical data processing and display devices. Several reports have already demonstrated the potential use of circularly polarised light for photonic devices in optical data storage^1^,^2^,^3^, highly efficient three-dimensional (3D) displays and liquid-crystal display (LCD) backlights^4^, effective spin sources in optical spintronics^5^,^6^, optical quantum information processing and communication^7^,^8^, and information carriers in quantum computation^9^ as well as the induction of exotic quantum phenomena such as the Floquet topological state^10^,^11^. However, wide-band reflective polarisers act as passive components in such devices^12^. The direct generation of circularly polarised light would be far more favourable in terms of energy efficiency and production costs.

Therefore, it is of great interest to construct CP-light-emitting devices. The use of wide-band reflective polarisers as passive components in organic light-emitting diodes (OLEDs) remains one means to engineer a CPL output but results in a relatively complex and thick device architecture, requiring an additional liquid-crystal cell^13^. In 1997, circularly polarised electroluminescence (CPEL) was first demonstrated in a polymer light-emitting diode fabricated using a chiral-substituted poly(*p*-phenylenevinylene) (PPV) derivative with low circular polarisation degree (g_PL_, emission dissymmetry factor)^14^. Later, several devices based on other chiral polymers or blends of small chiral molecules in the polymer were also reported with improved performances. However, for such fluorescence emitters, the internal quantum efficiency of the OLEDs is limited to 25% according to the spin statistics because only singlet excitons can emit light. Recently, phosphorescent polarized OLEDs are reported based on Pt(II) phosphors in mesogenic host-gest systems with well-designed nanostructures and linearly polarized phosphorescence was achieved with a quarter-wave plate^15^,^16^.

Phosphorescent iridium complexes play an important role in efficient OLEDs because of their high quantum efficiency and short lifetime of triplet excited states^17^,^18^,^19^,^20^,^21^,^22^,^23^. In most cases, the popular Ir(III) complexes used in OLEDs are small molecules with a six-coordinate octahedral structure, such as homoleptic complexes *fac*-Ir(ppy)_3_ (ppy = 2-phenylpyridine), heterogametic complexes Ir(ppy)_2_(acac) (acac = acetylacetonate) and FIrpic (iridium(III)bis[(4,6-difluoropheny)pyridinato-*N*,*C*^2^)^17^,^24^,^25^,^26^,^27^. However, all these complexes are used as racemic mixtures in OLEDs fabrication, and the ratio of two isomers is fixed as 1:1 according to the well-established synthesis route via μ-chloride dimer complexes. Although the racemic mixtures of Ir(III) luminophores emit light without net polarisation, enantio-enriched complexes emit circularly polarised light with a polarisation bias. Enantiomeric resolution of racemic, bidentate transition metal complexes into their optically active lambda (Λ, left-handed) and delta (∆, right-handed) isomers can be quite difficult. A few pure chiral Ir complexes have been separated and studied using electron circular dichroism (ECD) and circularly polarised phosphorescent photoluminescence (CPPPL) spectra^28^,^29^,^30^; however, none of them have been applied in OLEDs and other devices. If the chiral Ir(III) complexes are used as emitters, the circularly polarised phosphorescent electroluminescence (CPPEL) intensity and efficiency of the devices can potentially be significantly improved.

In this study, we demonstrated that nearly all the neutral Ir(III) complexes, including *fac*/*mer*- tris-cyclometalated Ir(C^N)_3_ and (C^N)_2_Ir(LX) with ancillary ligand, are racemic mixtures because of the configuration chirality, which can be separated through chiral preparative chromatography into stable Λ and ∆ optically active isomers. We selected the widely used *fac/mer*-Ir(ppy)_3_, Ir(ppy)_2_(acac) and Firpic complexes as examples. Additionally, we employed the ancillary ligands with a chiral carbon centre (*R*/*S*-edp, (*R*)/(*S*)-2-(4-ethyl-4,5-dihydrooxazol-2-yl)phenol) to obtain enantiomeric iridium complexes, namely Λ/Δ-Ir(dfppy)_2_(*R*-edp) and Λ/Δ-Ir(dfppy)_2_(*S*-edp) (dfppy = 4,6-difluorophenylpyridine), aiming to clarify the interactions between such different chiral factors on the chiroptical properties. Finally, OLEDs were fabricated with iridium isomers, and it is the first time that chiral iridium complexes have been used in OLEDs to directly generate circularly polarised light.

For the neutral Ir(III) complexes studied in this work, the Λ/Δ isomer ratios do not change with the variation of the temperature, solvent and concentration, suggesting that the chirality is an intrinsic property. The enantiomerically pure Λ and Δ isomers are separated with greater than 98% enantiomeric purity by chiral supercritical fluid chromatography with high yields (Table S1). Then, the isomers are either recrystallised at room temperature (*fac/mer*-Ir(ppy)_3_) or sublimed at approximately 250 °C (Ir(ppy)_2_(acac), FIrpic and Ir(dfppy)_2_(*R*/*S*-edp)). The unchanged absolute structures demonstrate the excellent chemical and thermal stabilities of these chiral isomers, guaranteeing the constant chirality during the fabrication of OLEDs at high temperature. The crystal structures of all the separated Λ/Δ isomers (Fig. 1 and Fig. S1, Table S2–S4, except Δ-*mer*-Ir(ppy)_3_) indicate that all the materials are crystallised into chiral space groups and that the absolute configurations are unchanged during the formation of the crystals, as reflected by the Flack parameters near zero.

It is interesting that the generation of the Λ and Δ isomers meets the weltanschauung of the old Chinese Taoism philosophy of the Eight Diagrams depicted in Fig. 2: Taiji generates Two Complementary Forces (One generates Two, for the racemic mixture can be divided into Λ and Δ configurations for normal Ir(III) complexes without chiral moiety). Two Complementary Forces generate Four Aggregates (two generates four, for Ir(III) complexes with one chiral (*R* or *S*) moiety in cyclometalated or ancillary ligand, Λ or Δ isomers can be further separated into Λ-*R*, Λ-*S*, Δ-*R* and Δ-*S*). Based on this regulation, we believe that if both the main ligand and ancillary ligand bare one chiral moiety (*R* or *S*), eight isomers can be finally achieved as Λ-*RR*, Λ-*RS*, Λ-*SR*, Λ-*SS*, Δ-*RR*, Δ-*RS*, Δ-*SR* and Δ-*SS* (Four Aggregates generate Eight Trigrams, four generates eight). Furthermore, Eight Trigrams determine myriads of phenomena (eight generates everything for the Ir(III) complexes with different chiral (*R* and *S*) moieties in the different positions of the cyclometalated and ancillary ligands).

The chiral structures of the iridium phosphor isomers can be further confirmed by the ECD spectra in CH_2_Cl_2_ (Fig. 3, Table 1). The ECD signals obtained for the Λ/Δ isomers of *fac*-Ir(ppy)_3_, Ir(ppy)_2_(acac) and FIrpic, which have only the configuration chirality, are perfect mirror images with opposite polarisation and almost equal ellipticity intensity as well as the dissymmetry factor *g* of absorption (Fig. S2), suggesting the opposite ground-state chirality. The weaker bands at approximately 400 nm of these spectra are dominated by metal-to-ligand charge transitions (MLCTs) and the strong band in the high-energy range stems from ligand-to-ligand charge transitions (LLCTs). Because the coordination configuration of the central iridium atom is the only chiral factor in these couples, the relatively high Δε of the R and L elliptically polarised light demonstrates that such structural chirality is indeed a strong chiral source.

The ECD spectra of Ir(dfppy)_2_(*R*-edp) and Ir(dfppy)_2_(*S*-edp) before chiral supercritical fluid chromatography are mirror-imaged because the chiral carbon atoms in the ancillary ligands are the only chiral source, and the chirality of configurations are smeared out because of the 1:1 ratio of Λ and Δ isomers in each racemic complex. However, the ECD spectra of the Λ/Δ isomers of Ir(dfppy)_2_(*R*-edp) and Ir(dfppy)_2_(*S*-edp) are only partial mirror-imaged because of the combined contributions from the chiral configurations and chiral ancillary ligands. Thus, Λ-Ir(dfppy)_2_(*R*-edp)/Δ-Ir(dfppy)_2_(*S*-edp) and Δ-Ir(dfppy)_2_(*R*-edp)/Λ-Ir(dfppy)_2_(*S*-edp) display two pairs of mirror-imaged ECD signals. Moreover, the ECD spectra of the Ir(dfppy)_2_(*R*-edp), Λ/Δ-Ir(dfppy)_2_(*R*-edp) and Ir(dfppy)_2_(*S*-edp), Λ/Δ-Ir(dfppy)_2_(*S*-edp) groups are also perfect mirror images, embodying the same intersection point at 402 nm. This interesting result can be explained by the Lambert-Beer principle. At this wavelength, there is no difference in absorption of R and L elliptically polarised light caused by chiral configuration, and the signals are simply increased by the chiral carbon atoms in ancillary ligands. In the remainder of the non-mirror-imaged range except this wavelength (402 nm), the ECD signals are affected by both chiral configurations and chiral carbon atoms. Finally, at 297 nm, all six lines conjunct into a single point on the horizontal axis, which indicates that both the chiral configuration and chiral carbon atoms have the same absorption of both R and L elliptically polarised light at the wavelength of 297 nm.

As examples, Fig. 4 presents the normalised UV-vis absorption and photoluminescence spectra of the Λ/Δ-*fac*-Ir(ppy)_3_, Λ/Δ-Ir(dfppy)_2_(*R*-edp) and Λ/Δ-Ir(dfppy)_2_(*S*-edp) in CH_2_Cl_2_, and no difference can be observed (Table 1, spectra of others were listed in Fig. S3). This finding is the main reason that iridium complexes with chiral configurations have not attracted much attention in previous studies. The normal photophysical properties are exactly the same between the racemic mixture and either isomer. The absorption spectra closely resemble those of typical Ir(III) complexes. The intense bands in the UV region are assigned to spin-allowed, singlet ligand-centred (^1^LC) transitions, whereas much lower intensity bands likely correspond to mostly spin-allowed ^1^MLCT and spin-forbidden ^3^MLCT transitions^31^ and are partially associated with ligand-centred transitions as well^32^. The emission spectra are relatively broad and featureless (an indication of predominant ^3^MLCT character). Indeed, cyclometalated iridium compounds have been well documented to emit from a mixed excited state, displaying characteristics of both ^3^LC and ^3^MLCT emission^33^.

The CPPPL spectra in CH_2_Cl_2_ with differential emission intensity (Δ*I*) as a function of wavelength of the iridium isomers (Fig. S4) exhibit the characteristic symmetry indicative of a predominance of either Δ or Λ isomer. The two peaks of each iridium isomer pair exhibit a “handedness” for the polarisation of the emitted light with completely positive or negative signals. To describe chiral luminescent compounds, the degree of CPPPL is defined by the emission dissymmetry factor (*g*_PL_ = 2 × Δ*I*/*I*, where Δ*I* = *I*_L_ − *I*_R_ is the difference in luminescence of left-and right-handed circularly polarised light, and *I* = *I*_L_ + *I*_R_ is the total emission intensity). In our cases, the overall *g*_PL_ values of most isomers in solution are on the order of 10^−3^ (Table 1, Fig. 5), similar to the typical values reported in the literature for transition metal complexes^34^,^35^,^36^. In addition, according to Fig. 5, all the complex isomers exhibit an almost constant *g*_PL_ value over a wide spectral range covering most of the emission band, further confirming the intrinsic chirality property. Among them, the *fac*-Ir(ppy)_3_ isomers exhibit the highest *g*_PL_ values of 3.15 × 10^−3^ (Δ) and −3.29 × 10^−3^ (Λ), and the *mer*-Ir(ppy)_3_ isomers exhibit the lowest g_PL_ values of 1.32 × 10^−4^ (Δ) and −2.97 × 10^−4^ (Λ) at the maximum emission wavelength because of their low internal quantum efficiencies. The complexes with ancillary ligands exhibit lower g_PL_ values than those of *fac*-Ir(ppy)_3_ isomers. When acac replaces one ppy to form Ir(ppy)_2_(acac) isomers, the g_PL_ values are decreased to 1.33 ×10^−3^ (Δ) and −0.915 ×10^−3^ (Λ). For Firpic isomers with a dfppy main ligand, the *g*_PL_ data are 1.66 ×10^−3^ (Δ) and −1.22  ×10^−3^ (Λ), slightly higher than that of Ir(ppy)_2_(acac) because the inclusion of substitutes at the 4 position of the phenylpyridine ligand results in an increased *g*_PL_ value^29^. The introduction of the chiral ancillary ligand can increase the *g*_PL_ values to 3.30 × 10^−3^ (Δ) and −2.61 × 10^−3^ (Λ) for Ir(dfppy)_2_(*R*-edp) and to 2.94 × 10^−3^ (Δ) and −2.90 × 10^−3^ (Λ) for Ir(dfppy)_2_(*S*-edp) isomers. Currently, the *g*_PL_ factors of the iridium Λ/Δ isomers are still low. Because the emission dissymmetry values are affected by the structure of the complexes, compounds incorporating greater helical twists should exhibit larger *g*_PL_ values by providing a circular path for electron flow. However, it is possible to determine the ligand positions that have effects on the chirality through computational calculations and to improve the *g*_PL_ factors of the iridium Λ/Δ isomers by molecular structure design, such as the introduction of electron-donating/-withdrawing or chiral groups to specific position of the ligands.

Because the CPPPL spectra reveal more information about the luminescent excited states, the monospecific CPPPL polarisation and nearly constant *g*_PL_ indicate that the chirality of the hybrid emission states of these iridium phosphor isomer pairs correspond to each other. Finally, it can be observed that the spectra outlines of the CPPPL are similar to the steady-state PL spectra, which demonstrates that for each isomer, the intensities of both left- and right-hand circularly polarised light exhibit the same change trend, together with the total emission intensity. Because the OLEDs with phosphorence iridium emitters always exhibit high performance, including high efficiency and brightness, these Δ/Λ isomers are still useful. Indeed, it is usually assumed that the sign and magnitude of the CPPPL signals are mainly determined by the Δ or Λ arrangement of the ligands. Remote asymmetric centres on the ligands have little direct effect on the observed spectra but contribute to the ECD and CPPPL signals because of their effect on chiral coordination. In the future, it will be possible to improve the *g*_PL_ values by introducing different chiral moieties to the different positions in both cyclometalated and ancillary ligands.

In order to investigate the circular polarized luminescent behaviour of iridium phosphors in vacuum processed devices, we fabricate the circularly polarised phosphorescent OLEDs using different Δ/Λ isomers, including the most widely researched homoleptic complex *fac*-Ir(ppy)_3_,heteroleptic complex Ir(ppy)_2_(acac) and heteroleptic complex Ir(dfppy)(*R*-edp) with chiral ancillary ligand. These research subjects are chosen to throw light on different classes of iridium phosphors. The general structure of the devices is ITO/TAPC (1,1-bis(4-(di-*p*-tolylamino)phenyl)cyclohexane, 60 nm)/Ir-complex (8 wt%): mCP (*N*,*N*’-dicarbazolyl-3,5-benzene, 20 nm)/TmPyPB (1,3,5-tri(*m*-pyrid-3-yl- phenyl)benzene, 40 nm)/LiF (1 nm)/Al (100 nm). The iridium complexes are always doped in a host material to avoid the undesirable serious triplet-triplet annihilation (TTA) resulting from the relatively long lifetime. Furthermore, such co-evaporation method usually guarantee amorphous film^37^,^38^, which is favourable for circular polarization behaviours, because measurement of circular polarization on crystalline samples are notoriously difficult. Since the chirality and CPL can be transferred from a chiral molecule to a nonchiral material^13^, we also measured the CPPPL spectra of the doped thin films (Table 1, Fig. S5) to test the effect of mCP on iridium Δ/Λ isomers. It can be observed that the effect of the mCP on the *g*_PL_ factors of iridium isomers is negligible. However, compared with the CPPPL spectra in solution, the emission peaks in the doped films are slightly blue-shifted.

The CPPEL spectra based on ΔI are depicted in Fig. S4. The CPPEL spectra also show completely positive or negative broad peaks, which correspond well to the results obtained for CPPPL in doped films (Figs S4 and S5) exhibiting a “handedness” for the polarisation of the emitted light. Comparing the CPPEL spectra peaks with the CPPPL spectra peaks in solutions, a more apparent blue shift can be observed and increases from homoleptic *fac*-Ir(ppy)_3_ (Λ: 8 nm, Δ: 7 nm) to heteroleptic Ir(ppy)_2_(acac) (Λ: 16 nm, Δ: 17 nm) and Ir(dfppy)_2_(*R*-edp) (Λ: 19 nm, Δ: 14 nm). This finding demonstrates that the CPPEL effect is an intrinsic property of the dissymmetric active layer and that the rigid solid-state host material matrix prevents molecule relaxation to a certain extent. The circularly polarised phosphorescent electroluminescence (CPPEL) spectra based on the g_EL_ factor are presented in Fig. 5. It can be found that the g_EL_ values for devices are almost ten times smaller than the corresponding g_PL_ values in solutions and mCP films, but these values still show obvious positive or negative signals. As we have demonstrated that host material mCP has little influence on g factors, such drastic decrease in g_EL_ should be attributed to device components and interfaces, for example, the back metal electrode on which the circular polarized electroluminescence is reflected and the polarization might be inversed may lead to a lower g_EL_ value. In addition, when the CPPEL pass through the hole transport layer and glass, the g_EL_ maybe also decreased. Because most of these iridium complexes have been extensively studied in devices before, we did not optimise the OLED performances, such as the luminance and efficiency. Compared with some former publications on different functional materials with circular polarized luminescence, such as helicene^39^ and rare earth complex^40^,^41^, although the emission dissymmetry factor (*g*_EL_) is only on the order of 10^−3^ and almost ten times smaller than g_PL_ in the corresponding solution samples for the iridium phosphors, the high device performances based on iridium complexes still make them useful in promoting OLED displays, such as 3D panels. In terms of practical application, a brightness of 100–200 cd/m^2^ is good enough for a 3D OLED display. This requirement indicates that for a device with a 10^−3^ order *g* factor, a brightness of 10^5^ cd/m^2^ can satisfy the requirement, which is easy to attain for OLEDs based on Ir(III) emitters. For example, with well-designed device architectures and specially developed host materials, the PhOLEDs using Ir(ppy)_2_(acac) can achieve a maximum brightness of 2.6 × 10^5^  cd/m^2^ 42, a power efficiency of 107.7 lm/W and external quantum efficiency (EQE) over 25%^43^. Meanwhile, for FIrpic, the power efficiency can reach 66.0 lm/W, the EQE is over 30% and the maximum luminance is also higher than10^5^ cd/m^2^ 44. We have doped the racemic *fac*-Ir(ppy)_3_ in the same OLED structure as the above, and a luminance over 2.2 × 10^5^ cd/m^2^ (11 V), a current efficiency over 73.4 cd/A and a EQE over 21% were obtained (Fig. S6).

In summary, the foregoing results demonstrate that nearly all the neutral iridium complexes prepared as racemic mixtures can be separated into stable optically active Λ and ∆ isomers. The CPPPL signals of Λ/Δ isomers have perfect mirror images with opposite polarisation and equal intensity, exhibiting a “handedness” for the polarisation. For the first example, the Λ/Δ iridium isomers are used as emitters in OLEDs, and the CPPEL spectra show completely positive or negative broad peaks. Our studies confirm that the chirality is an intrinsic property of the dissymmetric iridium complexes and that the Λ/Δ isomers have potential application for 3D OLEDs because most of the isomers always exhibit high device performances such as high efficiency and brightness, and 3D display technology based on circularly polarised light is the most comfortable for the eyes. Concerning the great development of device technology in the past decades, we believe that 3D OLED displays and other devices with chiral luminescent molecular systems will be a new research field in organic semiconductors and other related materials. For example, the pure organic materials always possess high g factor but low device efficiency. We can prepare chiral thermal active delay fluorescence (TADF) materials for efficient OLEDs.

## Additional Information

**How to cite this article**: Li, T.-Y. *et al*. Circularly polarised phosphorescent photoluminescence and electroluminescence of iridium complexes. *Sci. Rep*. **5**, 14912; doi: 10.1038/srep14912 (2015).

## Supplementary Material

Supplementary Information

## Figures and Tables

**Figure 1:**
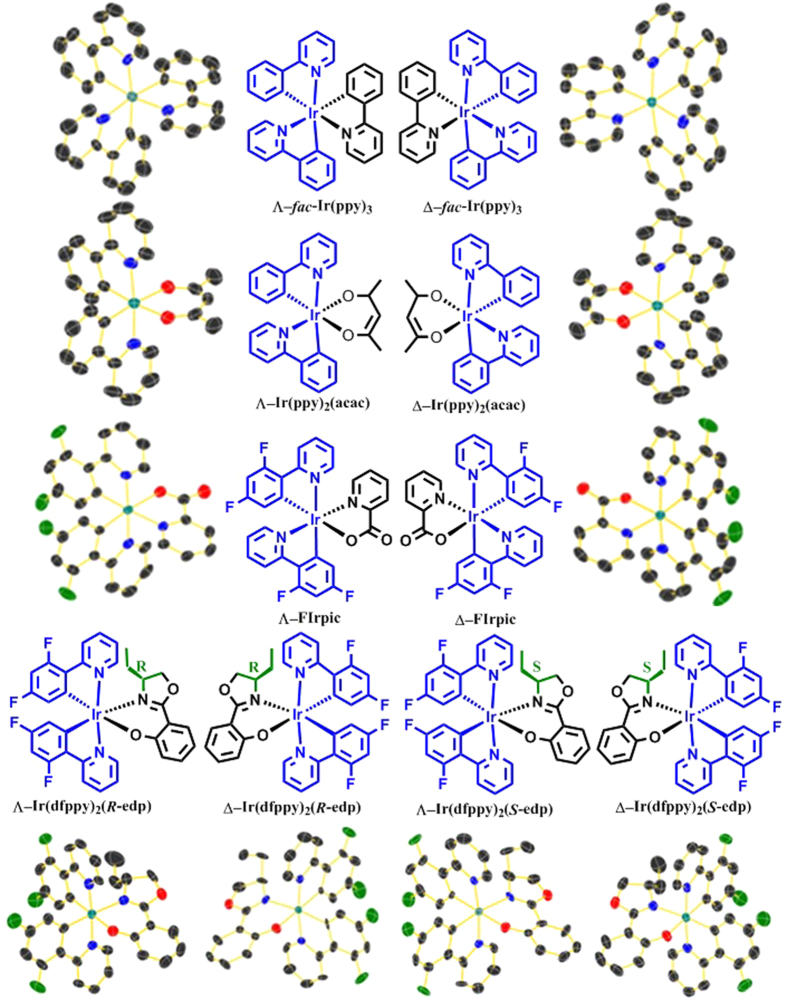
Chemical structures and crystallography ORTEP diagrams of Λ/Δ isomers. The thermal ellipsoids are depicted at 40% probability, and the hydrogen atoms are omitted for clarity. The following colours are used to represent the atoms: iridium (teal), carbon (black), oxygen (red), nitrogen (blue), fluorine (green).

**Figure 2:**
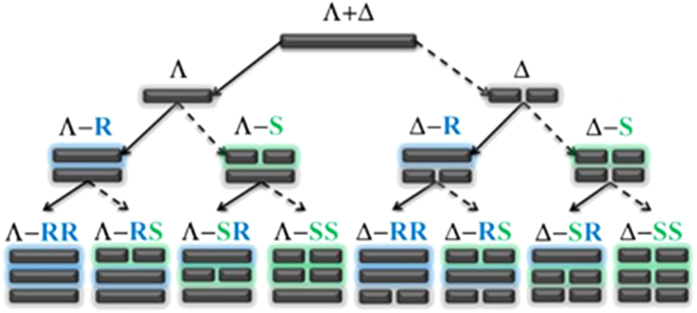
The generation of Λ/Δ isomers schematic diagram, similar to the weltanschauung of the old Chinese Taoism philosophy of the Eight Diagrams.

**Figure 3:**
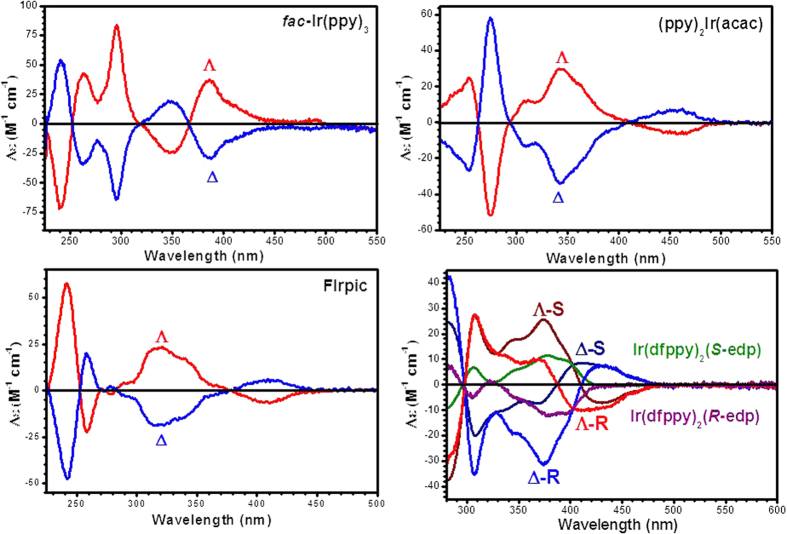
The electronic circular dichroism spectra of *fac*-Ir(ppy)_3_, Ir(ppy)_2_(acac), FIrpic and Ir(dfppy)_2_(*R*-edp) and Ir(dfppy)_2_(*S*-edp) isomers.

**Figure 4:**
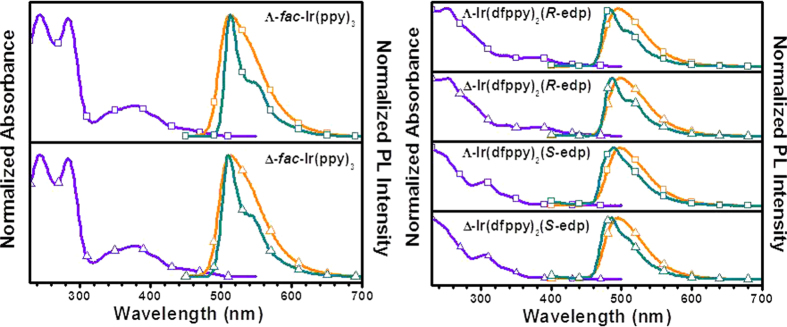
The absorption and emission spectra of *fac*-Ir(ppy)_3_, Ir(dfppy)_2_(*R*-edp) and Ir(dfppy)_2_(*S*-edp) isomers (absorption: purple, emission at room temperature/77 K: orange/teal).

**Figure 5:**
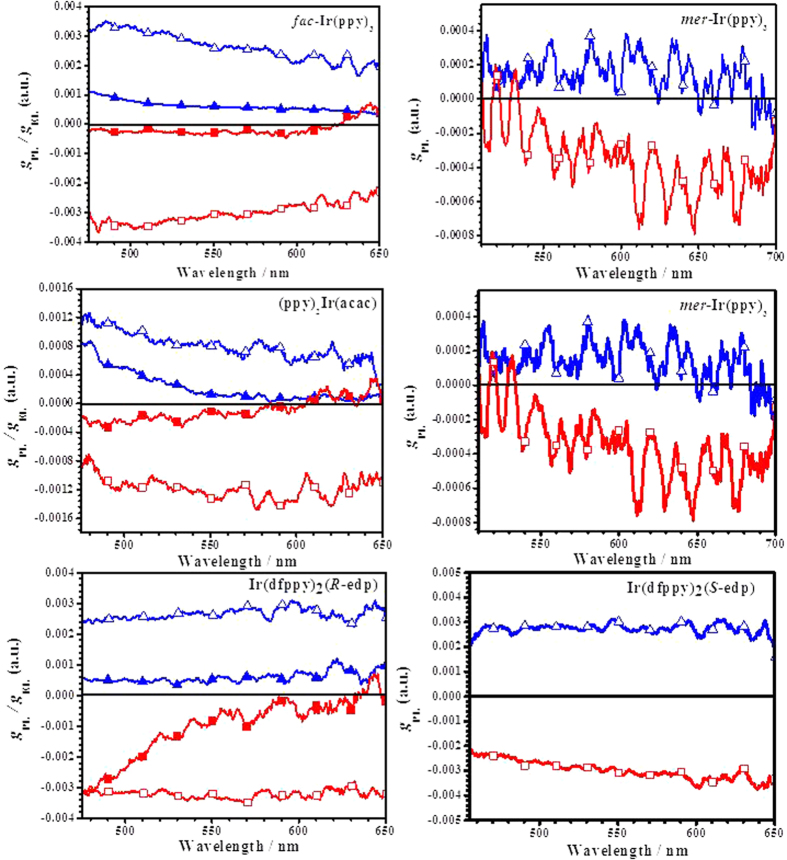
The plot of anisotropy factors g_PL_ for iridium isomers in DCM solutions (Delta: blue 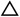, Lambda: red 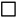) and g_EL_ for OLEDs based on different isomers of Ir(ppy)_3_, Ir(ppy)_2_(acac) and Ir(dfppy)_2_(*R*-edp) (Delta: blue 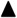, Lambda: red 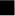).

**Table 1:** Photophysical data of all the iridium isomers.

		*λ*_PL_/nm	λ_ECD_ (ellipticity)/nm (mdeg)	*λ*_CPPPL_/nm		*g*_PL_/10^−3^		*λ*_CPPEL_ nm	*g*_EL_/10^−3^
Complex Configuration				solution	film	Solution	film		
*fac*-Ir(ppy)_3_	Λ	513	241(−71.5), 264(42.8), 296(84.0), 348(−24.5), 386(37.7)	529	515	−3.29	−3.30	522	−0.28
	Δ	514	241(54.4), 262(−34.1), 295(−64.2), 349(20.0), 388(−29.4)	523	516	3.15	3.22	515	0.68
*mer*-Ir(ppy)_3_	Λ	563	230(18.8), 256(−28.7), 283(11.0), 303(−27.0), 335(−10.2), 383(−10.3), 444(2.3)	623	–	−0.307	–	–	–
	Δ	563	232(−20.5), 256(32.3), 280(−7.9), 301(28.5), 332(10.2), 378(11.9), 447(−2.5)	586	–	0.188	–	–	–
Ir(ppy)_2_(acac)	Λ	521	253(25.1), 275(−51.7), 312(12.5), 342(29.9), 457(−6.7)	549	522	−1.33	−1,08	533	−0.31
	Δ	521	254(−27.0), 274(58.4), 309(−13.4), 343(−34.1), 462(7.6)	526	525	0.915	0.824	509	0.43
FIrpic	Λ	470, 491	214(58.1), 258(−22.5), 271(1.3), 278(−1.7), 320(23.3), 410(−6.8)	496	469	−1.66	−1.34	–	–
	Δ	471, 491	242(−47.6), 258(20.2), 272(−0.7), 278(2.2), 318(−18.8), 409(6.2)	486	472	1.22	1.31	–	–
Ir(dfppy)_2_(*R*-edp)	Λ	495	279(−31.5), 307(27.5), 370(10.1), 419(−10.4)	514	496	−3.30	−3.20	495	−2.6
	Δ	498	280(42.8), 307(−35.2), 344(−19.5), 374(−10.4), 432(7.9)	510	500	2.61	2.33	496	0.49
Ir(dfppy)_2_(*S*-edp)	Λ	497	281(−37.4), 307(27.0). 346(17.8), 374(25.6), 431(−7.1)	517	501	−2.94	−2.30	–	–
	Δ	495	281(24.5), 308(−20.1), 369(−7.3), 412(8.6)	514	491	2.90	2.83	–	–
