# Supplementary material for: Circularly polarised phosphorescent photoluminescence and electroluminescence of iridium complexes
Source: Sci Rep. 2015 Oct 8;5:14912. doi: 10.1038/srep14912 (PMC4597357; doi:10.1038/srep14912)
Supplement: Supplementary Information [file srep14912-s1.pdf]

Supporting Information

Circularly polarised phosphorescent photoluminescence and electroluminescence of iridium complexes

Tian-Yi Li[1], Yi-Ming Jin[1], Xuan Liu[1], Yue Zhao[1], Lin Shi[2], Zhiyong Tang[2], You-Xuan Zheng[1]* and Jing-Lin Zuo[1]*

[1] State Key Laboratory of Coordination Chemistry, Collaborative Innovation Center of Advanced Microstructures, School of Chemistry and Chemical Engineering, Nanjing University, Nanjing 210093, P. R. China, *e-mail: yxzheng@nju.edu.cn, zuojl@nju.edu.cn

[2] National Center for Nanoscience and Technology, 11 Beiyitiao, Zhongguancun, Beijing, 100190, P. R. China

Material and Synthesis

The iridium phosphorescent complexes, *fac*-Ir(ppy)$_3$, (ppy)$_2$Ir(acac) and FIrpic were bought from commercial sources. The meridional tris-cyclometalated complex (*mer*-Ir(ppy)$_3$) and the heteroleptic complexes with ancillary ligands baring a chiral carbon atom (*R*/*S*-edp) are synthesised according to the reported method (Scheme S1). The meridional tris-cyclometalated complex is obtained at a relatively low reaction temperature around 120 ^{o}C as a kinetically controlled product via μ-Cl dimer complex. For ancillary ligand *R*/*S*-edp, the chirality of the chiral C atoms can be maintained during the synthesis of both the ligands and the following iridium complexes. All the synthesized iridium complexes are purified by column chromatography (silicon gel, eluent = aceton/hexane 1:1 v/v) and recrystallization (diffusion of MeOH into DCM solutions). Since the structural chirality of the complexes is determined by the μ-Cl dimer complexes and no chiral selectivity is noticed during the formation of the dimer complex and the final products. All the commercial products and iridium phosphors synthesized according to the well-established method above are mixtures of equal molar of Λ and Δ isomers. Thus, until now, all the iridium phosphors used in the fabrication of the OLEDs are racemoids. In order to investigate the circular polarized phosphorescent electroluminescence behavior of each isomers, the enantiomer mixtures studied in this work are carefully separated by chiral synthetic HPLC method and sublimed afterwards. The detailed separation conditions can be found in Table S1 below.

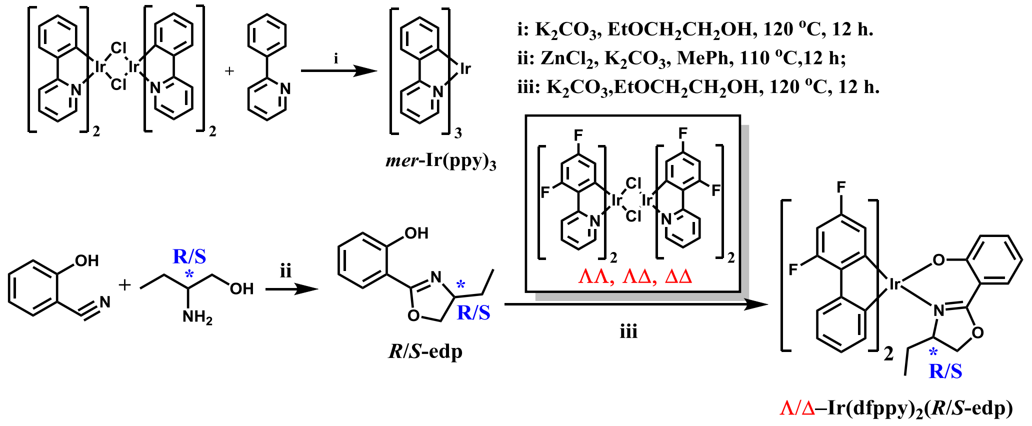

Scheme S1. The synthetic route of the *mer*-Ir(ppy)$_3$, chiral ligand *R*/*S*-edp and the racemic complexes Λ/Δ-Ir(*R*/*S*-edp).

Table S1 The detailed separation conditions for iridium isomers

Complex	*fac*-Ir(ppy)$_3$	*mer*-Ir(ppy)$_3$	Ir(ppy)$_2$(acac)	FIrpic	Ir(dfppy)$_2$(*R*-edp)	Ir(dfppy)$_2$(*S*-edp)
Column type	Daicel chiralpac IC	Daicel chiralpac IAS	Daicel chiralpac IC	Daicel chiralpac AD	Daicel chiralpac IF	Daicel chiralpac IC
Column size Inner diameter × length	5 cm × 25 cm	2.5 cm × 25 cm	5 cm × 25 cm	2.5 cm × 25 cm	2.5 cm × 25 cm	5 cm × 25 cm
Eluent (v/v)	Hexane:DCM= 50:50	Hexane:DCM=50: 50	Hexane:DCM=50: 50	Methanol	DCM:Methanol=90 :10	Hexane:DCM=50:5 0
Flow velocity (mL min^{-1})	13	10	60	20	25	13
Temperature (℃)	35	40	35	28	35	35
Sample volume (mL)	8	10	3	14	1	8
Sample concentration (mg mL^{-1})	0.5	0.3	5	4.9	10	0.5

Crystallography

Single crystals suitable for the study were grown either by evaporation of MeOH in the DCM solutions of the complexes (*fac*/*mer*-Ir(ppy)$_3$) or by slow sublimation at high vacuum ((ppy)$_2$Ir(acac), FIrpic and (dfppy)$_2$Ir(*R*/*S*-edp)).

The crystallographic data were collected by a Siemens SMART CCD diffractometer (Bruker Daltonic Inc.) at room temperature employing graphite-monochromated Mo Kα radiation (λ = 0.71073 Å). The crystals of *fac*-Ir(ppy)$_3$ obtained by solution method were measured in sealed tubes in mother liquor. The cell parameters were retrieved using SMART software and refined with SAINT on all observed reflections. Absorption corrections were conducted with SADABS supplied by Bruker. The final structures were resolved using SHELXTL software package. Initial atomic positions were located by Patterson method using XS program, and the structures of the complexes were refined anisotropically by the least-squares method. Hydrogen atoms were fixed in calculated positions and refined as riding atoms with uniform U_{iso} values. The absolute configurations of these chiral complexes were determined by the Flack factors.

Table S2. Crystal data of Λ/Δ-*fac*-Ir(ppy)$_3$ and Λ-*mer*-Ir(ppy)$_3$ isomers.

Complex	Λ-*fac*-Ir(ppy)$_3$	Δ-*fac*-Ir(ppy)$_3$	Λ-*mer*-Ir(ppy)$_3$	Δ-*mer*-Ir(ppy)$_3$
Formula	$C_{33}H_{24}N_3Ir$	$C_{33}H_{24}N_3Ir$	$C_{33}H_{24}N_3Ir$	
Formula weight	654.75	654.75	654.75	
Crystal system	Tetragonal	Tetragonal	Tetragonal	
Space group	$P4_32_12$	$P4_12_12$	$P4_32_12$	
a [Å]	9.7776(7)	9.808(5)	9.7776(3)	
b [Å]	9.7776(7)	9.808(5)	9.7776(3)	
c [Å]	30.953(4)	31.067(17)	31.0059(18)	
α [deg]	90.00	90.00	90.00	

β [deg]	90.00	90.00	90.00
γ [deg]	90.00	90.00	90.00
Volume	2959.2(5)	2988(3)	2964.2(2)
Z	4	4	4
R_1	0.0554	0.0258	0.0386
wR_2	0.1632	0.0611	0.1212
GoF	1.037	1.029	0.746
Flack index	0.09(3)	0.068(15)	0.00
CCDC No.	1033808	1033807	1033819

$R_1 = \Sigma||Fo|-|Fc|| / \Sigma|Fo|$, $wR_2 = [\Sigma[w(Fo^2-Fc^2)^2] / \Sigma w(Fo^2)^2]^{1/2}$

Table S3. Crystal data of Λ/Δ-(ppy)$_2$Ir(acac) and Λ/Δ-FIrpic isomers.

Complex	Λ-(ppy)$_2$Ir(acac)	Δ-(ppy)$_2$Ir(acac)	Λ-FIrpic	Δ- FIrpic
Formula	$C_{27}H_{23}N_2O_2Ir$	$C_{27}H_{23}N_2O_2Ir$	$C_{28}H_{16}N_3F_4O_2Ir$	$C_{28}H_{16}N_3F_4O_2Ir$
Formula weight	599.67	599.67	694.64	694.64
Crystal system	Trigonal	Trigonal	Orthorhombic	Orthorhombic
Space group	$P3_221$	$P3_221$	$P2_12_12_1$	$P2_12_12_1$
a [Å]	9.9437(14)	9.9527(6)	8.7505(4)	8.7512(3)
b [Å]	9.9437(14)	9.9527(6)	18.2045(7)	18.1997(5)
c [Å]	20.458(3)	20.4598(13)	30.6971(13)	30.7111(9)
α [deg]	90.00	90.00	90.00	90.00
β [deg]	90.00	90.00	90.00	90.00
γ [deg]	120.00	120.00	90.00	90.00
Volume	1751.8(4)	1755.15(19)	4890.0(4)	4891.3(3)
Z	3	3	8	8
R_1	0.0239	0.0189	0.0188	0.0274
wR_2	0.0554	0.0480	0.0401	0.0512
GoF	1.026	1.042	0.935	0.917
Flack index	0.006(14)	-0.004(12)	-0.006(4)	-0.006(5)
CCDC No.	1033806	1033805	1033810	1033809

$R_1 = \Sigma||Fo|-|Fc|| / \Sigma|Fo|$, $wR_2 = [\Sigma[w(Fo^2-Fc^2)^2] / \Sigma w(Fo^2)^2]^{1/2}$

Table S4. Crystal data of Λ/Δ-Ir(dfppy)$_2$(*R*-edp) and Λ/Δ-Ir(dfppy)$_2$Ir(*S*-edp) isomers.

Complex	Λ-Ir(dfppy)$_2$(*R*-edp)	Δ-Ir(dfppy)$_2$(*R*-edp)	Λ-Ir(dfppy)$_2$(*S*-edp)	Δ-Ir(dfppy)$_2$(*S*-edp)
Formula	$C_{33}H_{24}$ $N_3O_2F_4Ir$	$C_{33}H_{24}$ $N_3O_2F_4Ir$	$C_{33}H_{24}$ $N_3O_2F_4Ir$	$C_{33}H_{24}$ $N_3O_2F_4Ir$
Formula weight	762.75	762.75	762.75	762.75
Crystal system	Orthorhombic	Tetragonal	Tetragonal	Orthorhombic
Space group	$P2_12_12_1$	$P4_12_12$	$P4_32_12$	$P2_12_12_1$
a [Å]	10.5478(9)	9.0104(3)	8.9729(18)	10.5418(2)
b [Å]	15.1577(13)	9.0104(3)	8.9729(18)	15.1402(3)

c [Å]	36.343(3)	68.892(3)	68.592(14)	36.2904(8)
α [deg]	90.00	90.00	90.00	90.00
β [deg]	90.00	90.00	90.00	90.00
γ [deg]	90.00	90.00	90.00	90.00
Volume	5810.5(9)	5593.2(4)	5522.5(19)	5792.1(2)
Z	8	8	8	8
R_1	0.0527	0.0783	0.0641	0.0234
wR_2	0.1056	0.1773	0.1791	0.0472
GoF	1.017	1.143	1.388	0.953
Flack index	0.016(10)	0.090(11)	0.093(14)	-0.006(4)
CCDC No.	1033816	1033815	1033818	1033817

$R_1 = \Sigma||F\text{o}|-|F\text{c}|| / \Sigma|F\text{o}|$, $\text{w}R_2 = [\Sigma[\text{w}(F\text{o}^2-F\text{c}^2)^2] / \Sigma\text{w}(F\text{o}^2)^2]^{1/2}$

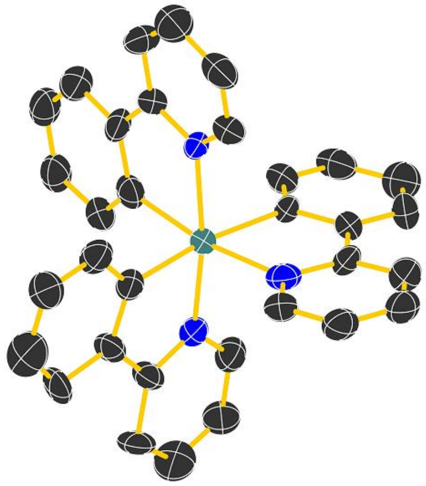

Figure S1. Chemical structures and crystallography ORTEP diagrams of Λ-*mer*-Ir(ppy)$_3$ isomers. The thermal ellipsoids are depicted at 40% probability, and the hydrogen atoms are omitted for clarity. The following colours are used to represent the atoms: iridium (teal), carbon (black), nitrogen (blue).

Spectral Measurements

The absorption profiles were measured on a Shimadzu UV-3100 spectrophotometer as CH_2Cl_2 solutions (1.0×10^{-5} M) at room temperature. The ECD spectra were recorded on a Jasco J-810 circular dichroism spectrometer with 'Standard' sensitivity at 100 nm min^{-1} scan speed with 0.5 nm resolution and respond time of 1 s. The unit in ECD spectra was transformed from mdeg (θ) into Δε by using the equation: Δε (cm^{-1} M^{-1}) = ellipticity (mdeg) / [32980 × b (cm) × c (M)], where b is the length of the light path and c is the concentration of the sample solutions. The magnitude of circular polarization in the ground state is defined as $g_{CD} = 2\times(\varepsilon_L - \varepsilon_R) / (\varepsilon_L + \varepsilon_R)$, where ε_L and ε_R represent the extinction coefficients for left and right circularly polarized light. Experimentally, the anisotropy factors g_{CD} is calculated as $\Delta\varepsilon/\varepsilon$ = [ellipticity / (32980)] / total absorbance. The photoluminescence spectra were obtained on a Hitachi F-4600 PL spectrometer as solutions CH_2Cl_2 (5.0×10^{-5} M) at room temperature and 77 K. The CPPPL spectra were measured on a Jasco CPL-200 spectrophotometer with 'Standard' sensitivity at 50 nm min^{-1} scan speed with 0.5 nm resolution and respond time of 8 s. The CPPPL signals were presented in ΔI and no corrections were applied on the CPPPL spectra. $\Delta I = I_L - I_R$, where I_L and I_R indicate the output signals for left and right circularly polarized light respectively. The magnitude of circular polarization in the excited state is defined as $g_{PL} = 2\times(I_L - I_R) / (I_L + I_R)$. Experimentally, the value of g_{PL} is defined as $\Delta I / I$ = [ellipticity / (32980 / ln10)] /

unpolarised PL intensity.

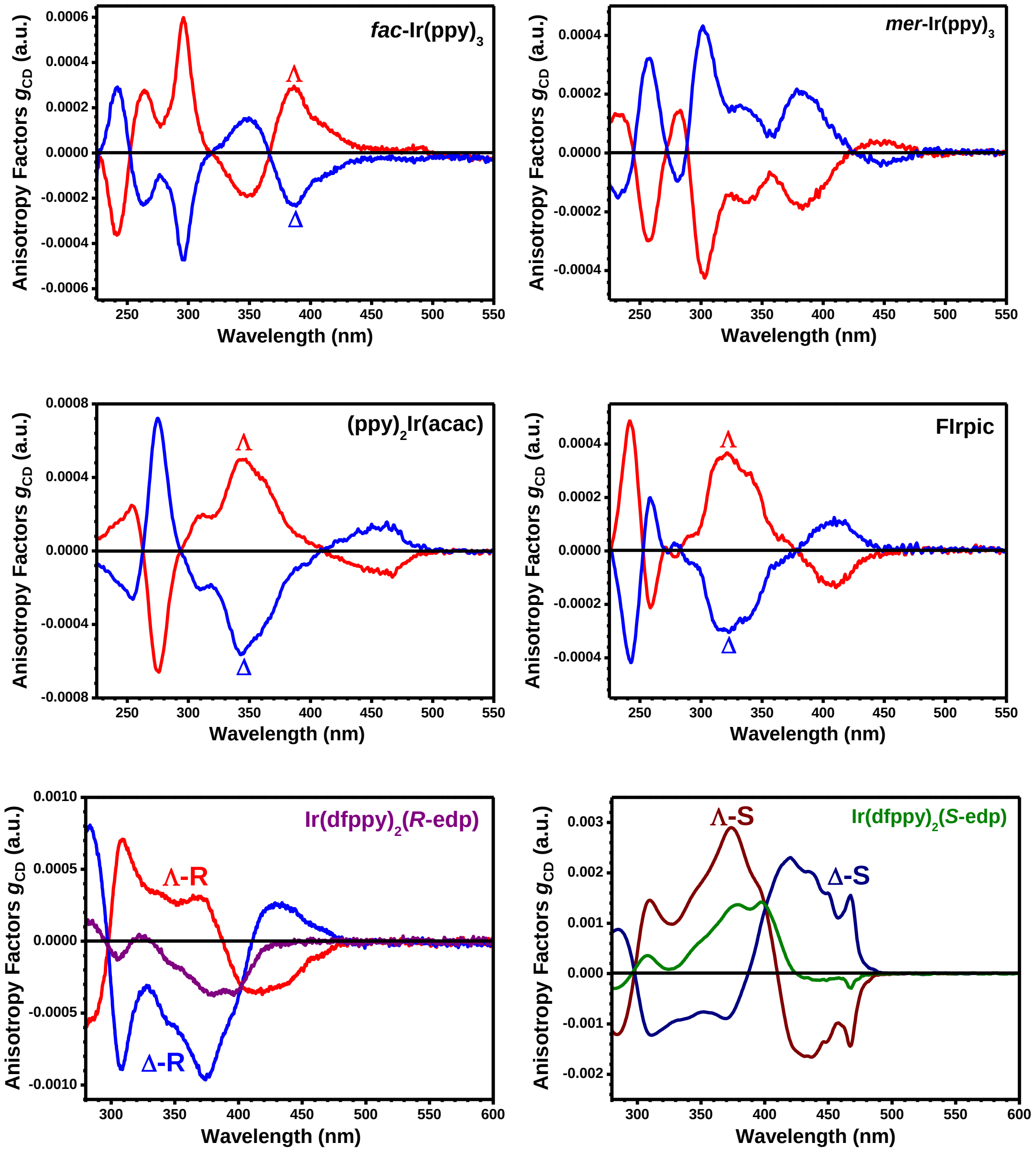

Figure S2. The ECD spectra based on anisotropy factors g_{CD}.

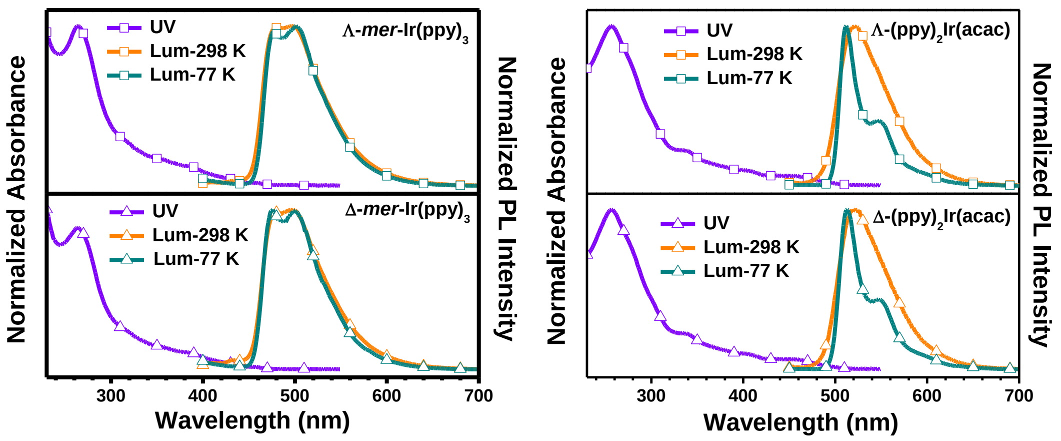

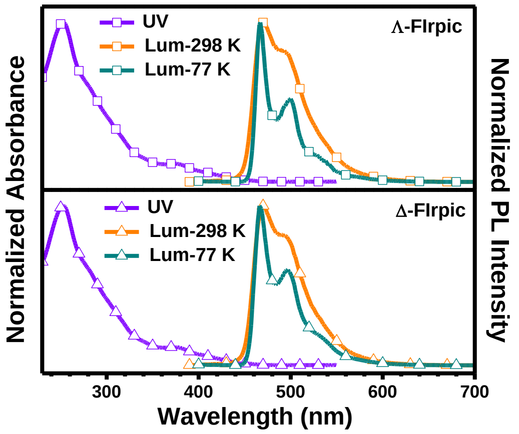

Figure S3. The UV-vis absorption and PL spectra of *mer*-Ir(ppy)$_3$, (ppy)$_2$Ir(acac) and FIrpic isomers (absorption: purple, emission at room temperature / 77 K: orange/teal).

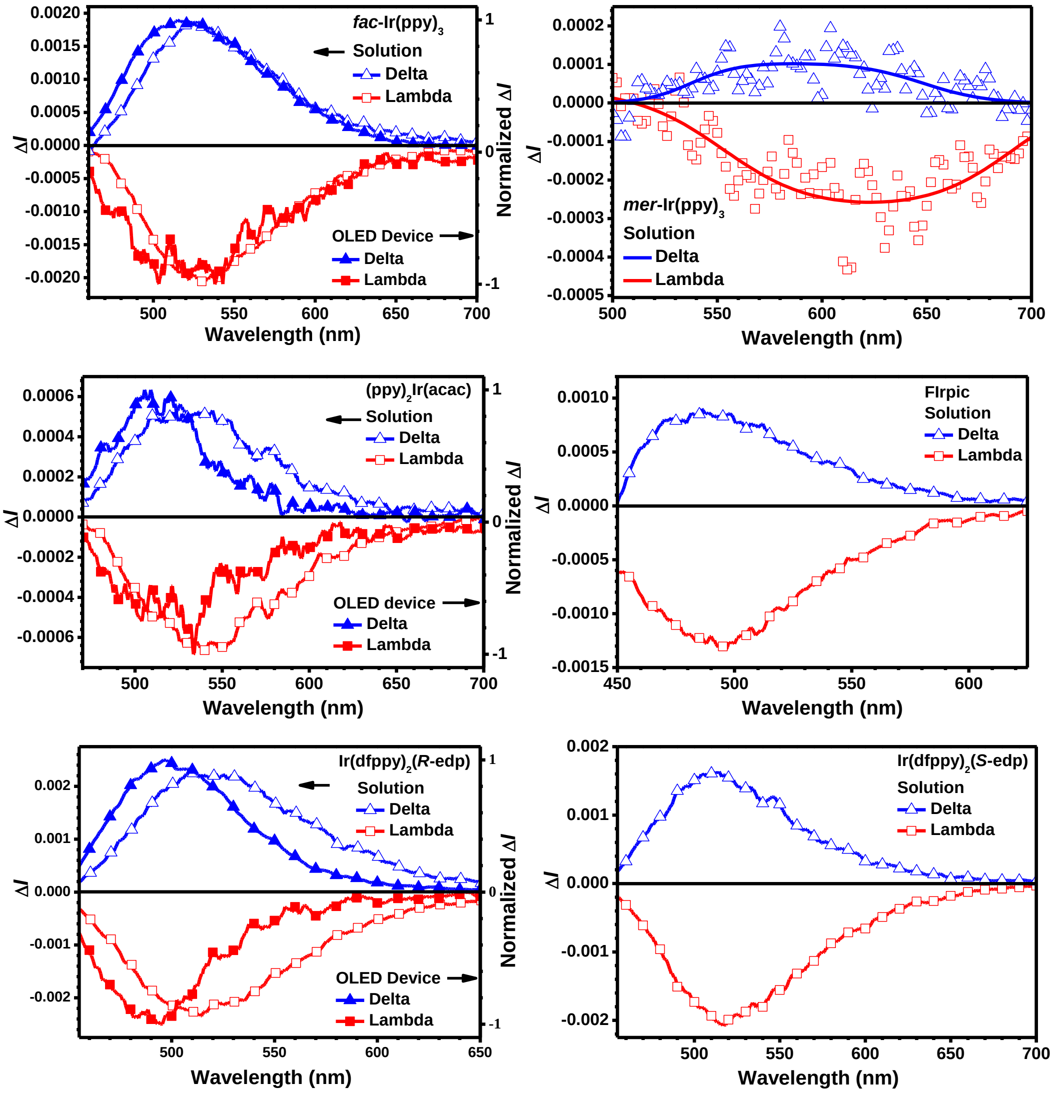

Figure S4. The CPPPL and CPPEL spectra of solutions and OLEDs based on Δ*I*.

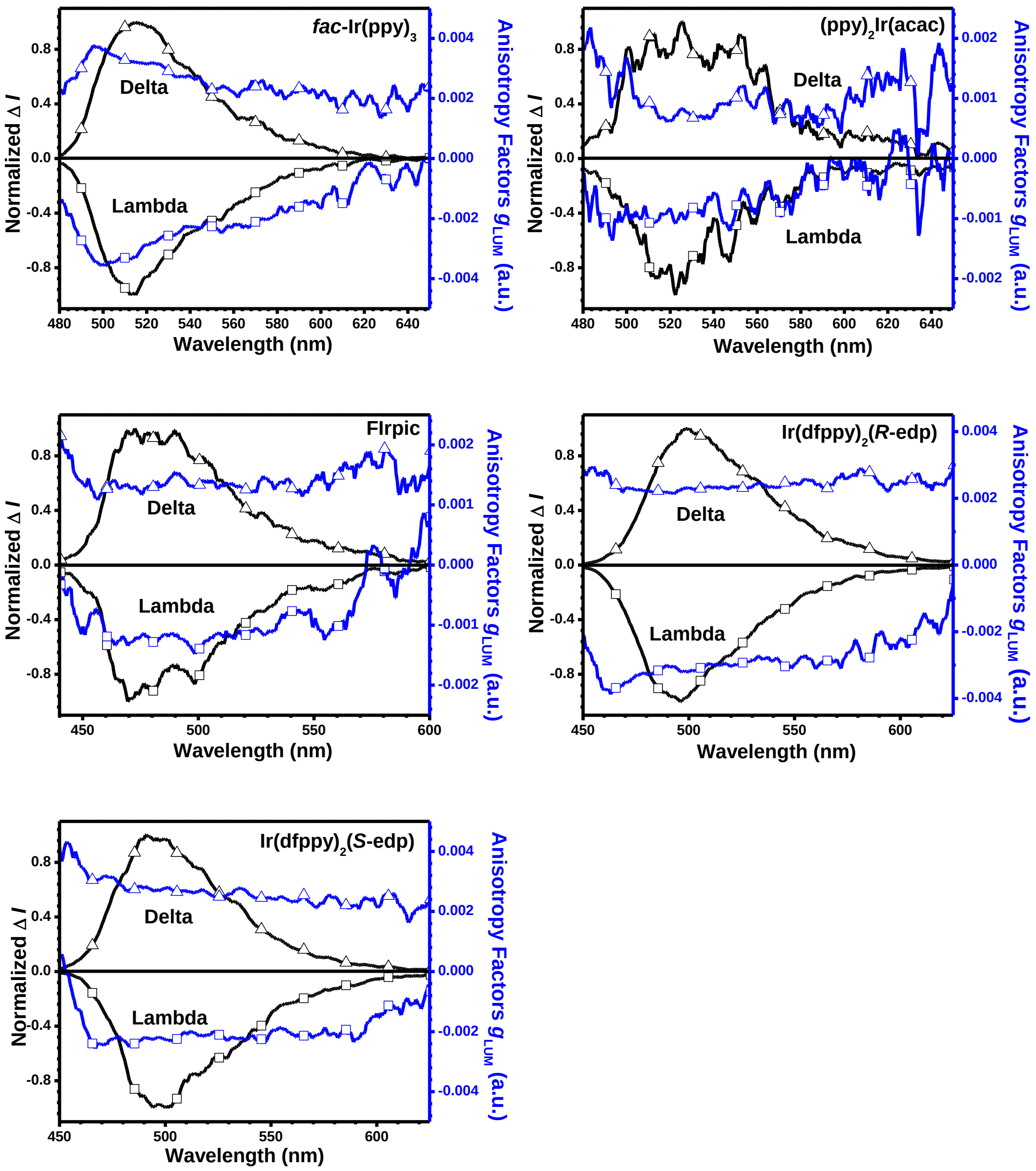

Figure S5. The CPPPL spectra of doped mCP thin films.

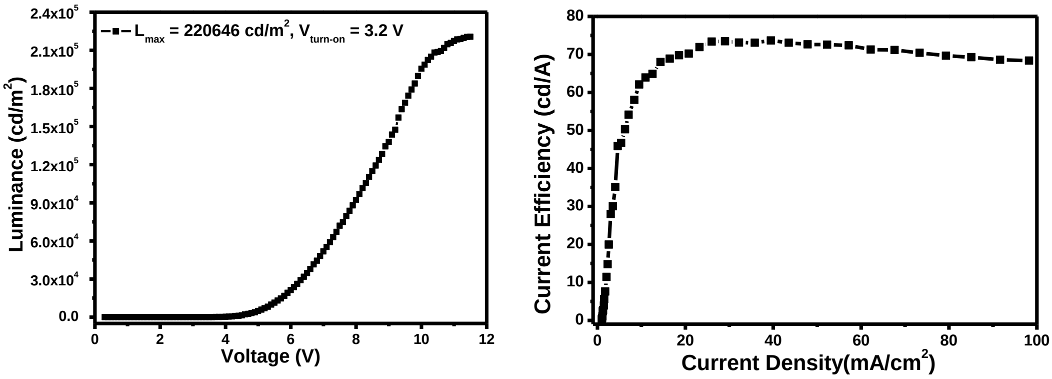

Figure S6. The luminance-voltage and current efficiency-current density curves of the device ITO / TAPC (1,1-bis[4-[*N*,*N*-di(*p*-tolyl)amino]phenyl]cyclohexane, 60 nm) / *fac*-Ir(ppy)$_3$ (8 wt%): mCP

(1,3-bis(carbazol-9-yl)benzene, 20 nm) / TmPyPB (1,3,5-tri[(3-pyridyl)-phen-3-yl]benzene, 40 nm) / LiF (1 nm) / Al (100 nm).
